# Association of farmers’ knowledge, attitude and practices with bovine brucellosis seroprevalence in Myanmar

**DOI:** 10.5713/ab.23.0273

**Published:** 2024-01-29

**Authors:** Su Su Hlaing, Satoko Kubota, Kohei Makita, Ye Tun Win, Hnin Thidar Myint, Hiroichi Kono

**Affiliations:** 1 Graduate School of Animal and Veterinary Sciences and Agriculture, Obihiro University of Agriculture and Veterinary Medicine, Obihiro 080-8555, Japan; 2 Department of Agro-environmental Science, Obihiro University of Agriculture and Veterinary Medicine, Obihiro 080-8555, Japan; 3 Department of Veterinary Medicine, Rakuno Gakuen University, 582 Bunkyodai Midorimachi, Ebetsu 069-8501, Japan; 4 Ministry of Agriculture, Livestock and Irrigation, Livestock Breeding and Veterinary Department, ZabuThiri 15011, Myanmar

**Keywords:** Bovine Brucellosis, Knowledge, Attitude, Practice, Custom, Myanmar

## Abstract

**Objective:**

This study aimed to identify the relationship between bovine brucellosis prevalence, farmers’ knowledge, attitude, practice (KAP), and social factors on migratory draft cattle and smallholder dairy farms in the central dry zone of Myanmar.

**Methods:**

This cross-sectional study was conducted on 54 migratory and 38 dairy cattle farms between August 2020 and February 2021. A structured questionnaire was used to identify farmers’ behaviors. Bulk milk was sampled and tested using indirect enzyme-linked immunosorbent assay (I-ELISA). STATA 17 was used for all the analyses.

**Results:**

Migratory cattle farms had a higher farm level brucellosis prevalence (14.8%) than dairy farms (2.6%; χ^2^ = 3.75; df = 1; p = 0.05). Only 2.8% of the farmers had knowledge about brucellosis, while 39.1% and 41.6% had attitudes and farm practices with respect to brucellosis, respectively in the study area. Socio-economic attribute of training in animal husbandry (p<0.01), raising system (p<0.01), practice of separating the aborted cow (p<0.01) were negatively associated to brucellosis. The overall farm level brucellosis prevalence was strongly associated with cattle herd size (p = 0.01), free movement grazing practices (p<0.01), practice of self-removal of placental debris without using personal protective equipment (p<0.01) and farmers’ attitudes towards eating cow placenta debris (p<0.01).

**Conclusion:**

Farmers had little knowledge of brucellosis. Attitudes and practices differed significantly between migratory and dairy farmers. Training and extension programs are necessary to make farmers aware of their KAP situation since livestock migration and the custom of eating cow placental debris contribute to the spread of brucellosis. Persistent efforts are required to reduce the adverse effects of brucellosis. Therefore, the study suggests that a feasible control intervention and public awareness campaigns need to be conducted regarding methods of preventing human exposure to brucellosis.

## INTRODUCTION

Myanmar is an agricultural country, with 23.46% of its gross domestic product coming from the agricultural sector, including livestock and fisheries [1]. There is an estimated total of 11.65 million cattle in Myanmar. Of these, 89% are owned by smallholder farmers, with an average of four cattle per farm [2]. Different production systems are used to raise cattle, including intensive, semi-intensive, tree-crop integration, and extensive systems. For example, cattle graze in and around villages, or cattle herds scavenge pastures for the resources (grazing and watering) they need to survive. Farmers seasonally migrate their cattle herds to common pastures, where they can graze freely for four months to a year. The cattle production sector in Myanmar is undergoing a significant transformation, and the demand for draft cattle is expected to reduce rapidly as mechanization increases. In addition, there is an increasing need to supply beef and milk to domestic and foreign markets in neighboring countries [2].

Livestock is a primary source of income for farmers. Hence, livestock diseases can negatively affect farmers’ livelihoods, local and national economies, and socioeconomic well-being. This has a negative effect on national food security and socioeconomic systems [3]. Brucellosis is a bacterial disease that is transmitted from livestock to humans through direct contact with animal birth and abortion materials as well as through the consumption of raw milk, meat, or blood [4]. The disease reduces milk productivity, increases the number of abortions and weak offspring, and is a major impediment to trade and export [5]. Despite its endemic nature in many developing countries, brucellosis remains neglected, underdiagnosed, and underreported [4].

The risk factors for *Brucella* infection include production systems such as grazing with shared pastures, agro-ecological zones, husbandry practices, contact with wildlife, and management factors [6,7]. Control of brucellosis in ruminants can be achieved through a combination of animal vaccination, removal of infected animals, and improvements in hygiene practices that minimize the risk of infection in disease-free herds [8]. Available evidence suggests that knowledge and awareness of brucellosis among veterinary practitioners are positively correlated with seropositivity in humans [9]. However, poor knowledge about brucellosis among livestock farmers has been reported in endemic zones where national disease control has not yet been implemented [10]. Knowledge, awareness, and practice (KAP) are well known to promote better awareness, which facilitates the implementation of hygiene measures [11].

Sociocultural conditions may influence the prevalence of brucellosis in animals and humans, particularly in rural areas [12]. Habits of living near animals, traditional consumption of raw milk and animal products, regular contact between people and animals, and improper handling of animal products have been reported to be associated with brucellosis in animals and humans [13]. Kristensen and Jakobsen [14] pointed out that social epidemiology refers to a holistic approach that integrates animal husbandry and an understanding of farmers’ behaviors to control disease incidence.

As bovine brucellosis is a neglected zoonosis in Myanmar, there is no mandatory vaccination program, and the development of a disease control plan is still underway [15]. According to the World Organization for Animal Health [16], the status of bovine brucellosis in Myanmar has not been clarified by epidemiological reports, and it has been suggested that the disease situation and risk factors should be better understood by conducting socioeconomic studies, such as KAP surveys, to ensure regional brucellosis control [17]. A recent study in Myanmar showed that the prevalence at the herd level was 12.3% and that there was a lack of appropriate husbandry practices and brucellosis knowledge among traditional draft cattle farmers [18].

Despite previous epidemiological surveys (conducted mostly on dairy farms), little is known about farmers’ behaviors and social customs related to bovine brucellosis, although their importance has been highlighted [16]. The objective of this study was to clarify the relationship between the prevalence of bovine brucellosis, farmers’ KAP, and social customs among seasonal migratory draft cattle farms and smallholder dairy farms in the central dry zone (CDZ) of Myanmar.

## MATERIALS AND METHODS

### Study area

**Figure 1:**
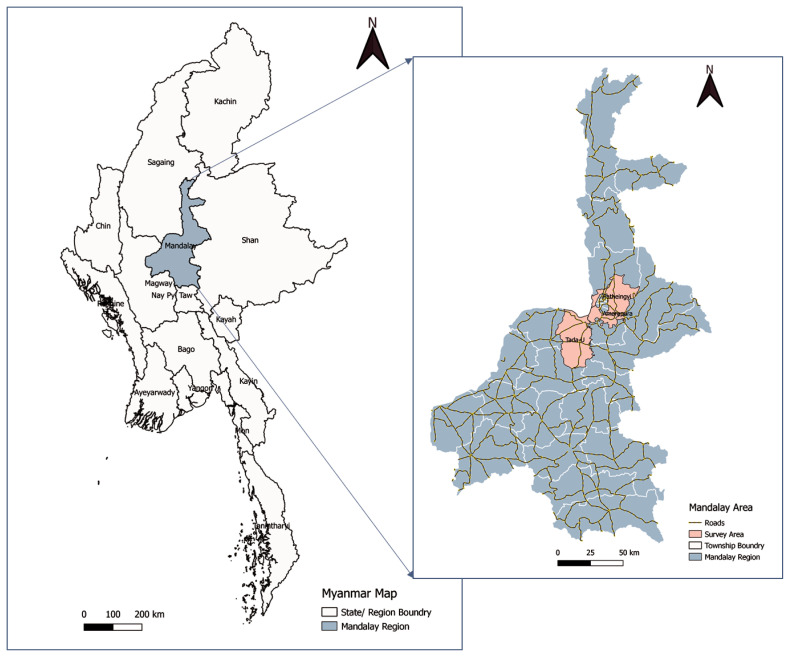
Myanmar Map showing the study area, Mandalay Region.

The study areas included herein were Amarapura, Tada-U, and Patheingyi townships in the Mandalay region of Myanmar (Figure 1). The Mandalay region accounts for more than half (52.77%) of the country’s cattle population [2]. In this region, breeding draft cattle is the highest priority of livestock programs (23% of households raise draft cattle). In contrast, dairy farming for commercial milk production is still at an early stage of development, raising three to five cows as smallholders (only 0.30% of households raise dairy cattle) [2]. Livestock migration is a common seasonal practice to cope with local environmental constraints. The central dry zone is characterized by migratory herds from pastures and water-scarce areas to abundant places from 4 months to a year. Livestock in Myanmar is mainly reared on ‘backyard farms’, with feeding provided in traditional ways such as grazing common in fallow areas within and around villages or scavenging in the village environment and utilizing standing crop residues and by-products [2].

### Field survey and sampling methods

**Figure 2:**
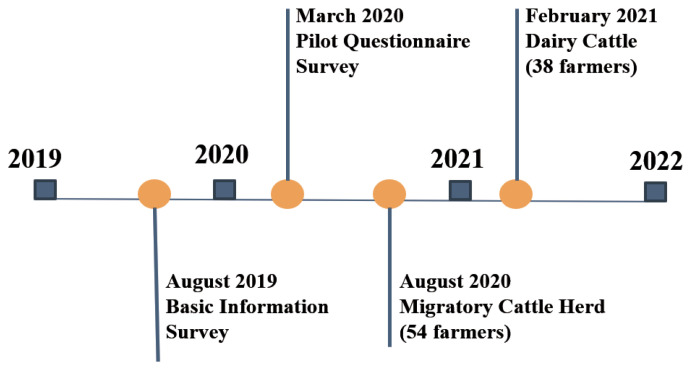
The study timeline.

A cross-sectional study of migratory and dairy farms involving blood sampling and interviews, using a structured questionnaire, was designed based on the preliminary findings from a basic information survey conducted in August 2019 (Figure 2). The questionnaire consisted of two parts: i) demographic characteristics, including livestock management practices, and ii) KAP associated with brucellosis. It was prepared with close-ended and open-ended questions, initially in English, and then translated into Burmese language to facilitate the interviewing of the farmers. In March 2020, a pilot study was conducted on 10 purposively selected farmers from migratory and dairy farms to assess the clarity of the questionnaire instructions and layout and the time required for completion (Figure 2).

Field surveys were conducted on 54 migratory cattle farms in Amarapura and Tada-U in August 2020, and on 38 dairy farms in Patheingyi in February 2021. Systematic random sampling techniques were used to select dairy farms and purposive sampling from the migratory farms. To compare the prevalence of farm level brucellosis prevalence, the sample size was calculated using Formula [19].


(Formula) $$\text{n}=\frac{{\left(z_{\alpha }\sqrt{pq}-z_{\beta }\sqrt{p_{1}q_{1}+p_{2}q_{2}}\right)}^{2}}{{\left(p_{1}-p_{2}\right)}^{2}}$$

where, *n*, sample size; *z_α_*, standard normal deviate at 1.96, corresponding to a 95% confidence interval, *z_β_*, standard normal deviate at −0.84, corresponding to a power of 80%; *p*_1_,18.18%; *p*_2_, 7.7%; *p* = mean of *p*_1_ and *p*_2_; *q* = 1–*p*. The estimated prevalence, *p*_1_, was based on 18.18% [20] and *p*_2_ was set according to our assumption of a 7.7% difference in prevalence. The sample size in both areas (for migratory and dairy farms) was calculated as 100; however, owing to the occurrence of coronavirus disease 2019 (COVID-19), only 54 and 38 farmers, respectively, could be visited.

Data were collected from individual farmers through face-to-face interviews, using a structured questionnaire. Questionnaires were administered by veterinary advisors from the Livestock Breeding and Veterinary Department (LBVD), Myanmar. Milk samples were aseptically collected from a bulk milk tank during visits to each cattle farm. After milk samples were collected, they were transported to the laboratory in cool boxes and stored at 4°C for at least 24 hours. All milk samples collected were analyzed using a commercial indirect enzyme-linked immunosorbent assay (I-ELISA) kit for *Brucella abortus* antibody according to the manufacturer’s protocol (I-ELISA; ID.vet, Grabels, France). I-ELISA was found with the best sensitivity and specificity estimates 96.8 (95% PI: 92.3 to 99.1) and 96.3 (95% PI: 91.7 to 98.8) [21]. The seropositivity value (S/P) was calculated to evaluate test results.

### Data analysis

Data from the survey were digitized in a Microsoft Excel spreadsheet and organized data were transferred to Stata version 17. To analyze the scores for Brucellosis knowledge, attitudes, and practices-related questions were addressed. To assess knowledge of brucellosis, seven objective questions on clinical signs of brucellosis were included, followed by one subjective question “Know brucellosis disease (KNOW)” (Table 2). True, false, and do not know responses were given to determine knowledge scores. A correct response received one point, while incorrect answer received a zero point. Five basic management questions were asked to estimate the score of farming practices, and five behavioral questions were asked to estimate the score of attitudes, with yes or no responses. Descriptive statistics (Table 1) provide an overview of the main social and farm elements of mean, chi-square, and probability values. In this study, we employed the Probit model to determine socioeconomic and demographic factors influencing *Brucella* seropositivity at the farm level. The Probit model is a statistical probability model with two dependent variable categories [22]. The cumulative normal probability distribution is used in probit analysis. The probit analysis provides statistically significant results on whether demographics increase or decrease the likelihood of *Brucella* seropositivity at the farm level. The Probit model was employed to determine the relationship between *Brucella* seropositivity (*BRUCELLA*) as dependent variable and Farmers’ age (*AGE*), primary education (*EDU*), experience (*EXP*), common grazing (*GRAZING*), cattle herd size (*CATTLE*), raising system (*RAISE*), training (*VET TRAIN*), as well as their knowledge of brucellosis (*KNOW*), the attitude of eating cow placental debris (*EAT*), practice of self-removal of placental debris by bare hands (*HAND*) and practice of separation of aborted cows as independent variables. Tables 1 and 2 summarize the variables used in this study.

## RESULTS

### Brucellosis prevalence at farm level

The overall *Brucella* seropositivity at the farm level was 9.8% (9/92; 95% CI: 0.05 to 0.18). There was a significant difference in farm-level prevalence between migratory farms (8/54, 14.8%) and dairy farms (2/38, 2.6%) in the study area (*χ*^2^ = 3.75; df = 1; p = 0.05) (Table 1).

### Socio-economic characteristics

The study interviewed 92 farmers, of whom 58.7% (n = 54) were migratory and 41.3% (n = 38) were dairy farmers. Table 1 presents the descriptive statistics for all variables. The mean age of the respondents was 44 years (range: 23 to 90 years), and 53.3% of the household heads had achieved a primary level of education. In terms of herd management, dairy farmers had more farming experience (20 years) than migratory cattle farmers (15 years) and maintained an average of 11 animals per farm from their parents’ time (p = 0.01). A large proportion of migratory farmers were observed to raise cattle in an extensive system (88.9%; p<0.01), whereas 62.9% fed forage by grazing (p<0.01).

### Farmers’ knowledge, attitudes, and practices of brucellosis

Of the 92 respondents who were asked about their knowledge of bovine brucellosis, a large majority (80.4%, 74/92) had never heard of the disease (Table 2). Farmers who had heard of brucellosis (19.6%, 18/92) were unaware that *Brucella* infections could cause decreased milk production (p = 0.07).

The average percentage of farmers’ attitude levels was 39.1%, with 28.6% and 49.5% for migratory and dairy farmers, respectively, which was statistically significant (p = 0.04) (Table 2). A total of 81.5% (75/92) of the respondents believed that they should report abortion cases to their veterinarians, and 68.4% (26/38) of dairy farmers collected new information about bovine diseases. Dairy farmers accounted for 21.1% of the respondents who adhered to the attitude that placental debris should be eaten (*EAT*), whereas migratory farmers had a less favorable attitude towards eating placental debris (p<0.01).

The average practice level of farmers was 41.6%. In comparison to migratory farmers, dairy farmers practiced proper farm practices such as separating diseased animals (*SEPARATE*) (60.5%; p = 0.09) and animals that had aborted (52.6%; p = 0.10). Furthermore, dairy farmers both sold milk (57.9%; p<0.01) and consumed raw milk (42.1%; p<0.01) from cows that had aborted. There was a significant difference between farmers in the research area (65.2%) who used their bare hands to remove placental debris (p<0.01).

### Factors associated with farm-level bovine brucellosis seropositivity

The *Brucella* seropositive herds were characterized by larger cattle herds (coefficient = 0.075; p = 0.011), more extensive system (coefficient = 30.899; p<0.01), more common grazing practice (coefficient = 31.359; p<0.01), not receiving veterinary training (coefficient = −4.626; p<0.01), more practice of removing placental debris by bare hand (coefficient = 6.406; p<0.01), less practice of separating aborted cows on farm (coefficient = −8.482; p<0.01) and attitude towards eating cow placental debris (coefficient = 4.035; p<0.01).

## DISCUSSION

This study was conducted in three areas of the CDZ of Myanmar, which represents over half of the cattle population in the country, and has the potential to be generalized to the other 57 areas in the CDZ, which have similar climate conditions [2]. On average, the knowledge score of farmers for brucellosis was only 19.6%, with little difference between migratory and dairy farms. This is essentially in line with a previous study [23], which found that 15% of farmers in Tajikistan knew about brucellosis. In contrast, only 2.6% of farmers in Sri Lanka [12] and 4.8% of farmers in India [24] were aware that brucellosis is a zoonotic disease. Farmers’ attitudes towards the spread of brucellosis were undesirable in this study, as indicated by the selling of cows that had aborted to other farms, as well as their lack of involvement in training programs. Brucellosis transmission-related practices, such as self-removal of placental debris by bare hands, selling milk from cows that had aborted, and consuming milk from cows that had aborted, were not satisfactory. There was a higher likelihood of seropositive animals being found on farms that practiced an extensive rearing system. There was no association between farmers’ knowledge and practices and the presence of *Brucella* seropositivity at farm level.

Farmers’ knowledge about brucellosis is significantly related to training, and the main source of farmers’ knowledge is veterinary advice on animal health issues [6]. The current study found that dairy farmers were more likely to attend training provided by LBVD and dairy cooperatives, whereas backyard and migratory cattle farms did not have farm cooperatives, making such training more difficult. The farmers interviewed had considerable experience in cattle management through animal husbandry training conducted by the LBVD; however, a large majority of the farmers had not received any information about brucellosis. This could be because the government focused on improving livestock productivity and increasing the accessibility of animal health services nationwide through veterinarians and community animal health workers. At the farm level, *Brucella* infection was present on dairy and migratory farms in the study area at 2.6% and 14.8%, respectively. The overall level was 9.8%, which is lower than the previously reported 26% in domestic cattle herds [25] and 24.1% in dairy cattle in Thailand [26]. Although the prevalence in this study was lower than that in previous local reports, it was higher at the farm level than the prevalence of 4.1% reported previously [27]. Interestingly, migratory farms had a higher prevalence of brucellosis than dairy farms in the study area, despite the dairy farmers having substantially more farming experience. Migratory cattle herds are more likely to come in contact with other cattle, share pastures with other herds, and are often located near livestock markets and along cattle trade routes. These findings can be linked to an increase in the number of cattle and frequent contact among animals in large herds, which is one of the determinants of *Brucella* infection, particularly during abortion or calving, and has a strong relationship with the occurrence of brucellosis.

The research in Uganda has demonstrated that the consumption of raw milk and animal products is a significant risk factor for disease transmission from animals to humans [28]. Studies in Pakistan have revealed that approximately 66% of households consume raw milk; however, only 3.0% knew that brucellosis could be transmitted through raw milk [29]. According to the results of this study, *Brucella* seropositivity is highly correlated with farmers’ attitudes towards consuming cow placental debris as well as how they handle aborted fetuses without personal protective equipment; a lack of veterinary training; not separating aborted cows from other cows on the farm. Very few studies have reported on the traditional customs of eating cooked placentas among farmers in rural communities. People living in Myanmar believe that eating cooked cow placental debris is healthy and do not consider the risk of brucellosis. In particular, those living in rural societies maintain their longstanding beliefs and traditionally consume cow placental debris. In the case of the indirect causal pathway that bovine brucellosis seropositivity could be associated with the custom of humans eating cows’ placenta, farmers have the practice of handling the vaginal discharges and blood with no gloves and can properly unprotected contact with the other cattle and contaminated with feed and water before cooking the placenta. Furthermore, a lack of knowledge for brucellosis disease as a result of limited access to veterinary training as well as farmer behavior such as eating cooked cow’s placental debris and cattle migration practices highlighted the significance of human exposure to brucellosis.

## CONCLUSION

This study found that migratory cattle farms had a higher prevalence of brucellosis than dairy farms in the study area. Farmers had low levels of knowledge about brucellosis. The level of practice and attitude differed significantly between migratory and dairy farmers. The custom of eating cow placental debris is an indirect causal pathway contributing to the prevalence of brucellosis in cattle. Considering that cattle migration and the attitude of eating placental debris are major factors in the spread of brucellosis, it is imperative for farmers to be educated about their KAP through government animal health training, particularly focusing on migratory farms. Therefore, persistent efforts are required to reduce the adverse effects of brucellosis. In light of the findings of the study, a feasible control intervention as well as public awareness campaigns regarding methods of preventing human exposure to brucellosis are recommended.

## Figures and Tables

**Table 1:** Farmer and farm characteristics in the study areas

Variables	Definition	Total farms (n = 92)			χ^2^ (df = 1)	p-value

			Migratory farm (n = 54)	Dairy farm (n = 38)		
**Farmers’ characteristics**						
Farmer age (***AGE***)^1)^	Farmer’s average age in years	44	40	49	47.18	0.12
Education (***EDU***)^1)^	1: Primary; 0: Other level	53.3%	53.7%	52.6%	0.01	0.92
Experience (***EXP***)^1)^	Average years working in livestock farming	17.5	15	20	34.15	0.20
Common grazing (***GRAZING***)^1)^	1: cattle grazing in common pasture; 0:no grazing	46.8%	46.7%	1.1%	50.60	<0.01^***^
Migration (***MIGRATE***)^1)^	1: Migratory; 0: Not migratory	58.7%	100%	0%	92	<0.01^***^
**Farm characteristics**						
Cattle herd size (***CATTLE***)^1)^	Average cattle heads in farm	19	28	11	61.89	0.01^**^
Feeding system	1: Forage; 0: Concentrate	38.0%	62.9%	2.6%	34.4	<0.01^***^
Raising system (***RAISE***)^1)^	1: Extensive; 0: Intensive	52.2%	88.9%	0%	70.63	<0.01^***^
Training (***VET TRAIN***)^1)^	1: Attend animal husbandry veterinary training; 0: Other or no training	1.1%	0%	2.6%	1.44	0.23
*Brucella* sero-positivity at farm level (***BRUCELLA***)^1)^	1: Positive; 0: Negative	9.8%	14.8%	2.6%	3.75	0.05^*^
Abortion case (***ABORTION***)	Average abortion case in cows in past 1 year	7.6%	7.6%	0%	5.33	0.02^**^

1) Bold text in brackets indicates the variable used in the Probit model (Table 3).

***, **, and * Indicate significance at the 1%, 5%, and 10% levels, respectively.

**Table 2:** Knowledge (K), Attitude (A) and Practice (P) comparisons between migratory and dairy farmers (n = 92)

Variables	Total farms (n = 92)			p-value

		Migratory farm (n = 54)	Dairy farm (n = 38)	
**Knowledge (K)**				
Know brucellosis disease^1)^ (***KNOW***)^2)^	18 (19.6%)	10 (18.5%)	8 (21.1%)	0.76
Reduced milk production (True)	4 (4.34%)	1 (1.9%)	3 (7.9%)	0.07^*^
Abortion of cow (True)	4 (4.34%)	1 (1.9%)	3 (7.9%)	0.16
Swelling of joints (arthritis) (True)	1 (1.9%)	1 (1.9%)	0 (0.0%)	0.39
The cow will be blind (False)	2 (2.2%)	2 (3.7%)	0 (0.0%)	0.39
Blisters in the mouth (False)	1 (1.9%)	1 (1.9%)	0 (0.0%)	0.39
Transmission from cow to cow (True)	2 (2.2%)	1 (1.9%)	1 (2.6%)	0.36
Transmission from cow to human (True)	3 (3.3%)	1 (1.9%)	1 (2.6%)	0.36
**Average**	2.8%	2.6%	3.0%	0.37
**Attitude (A)**				
Cows that had aborted should not be sold to other farms	21 (22.8%)	10 (18.5%)	11 (28.9%)	0.24
Collect new information about cattle diseases	47 (51.1%)	21 (38.9%)	26 (68.4%)	0.01^***^
Participate in training about cattle diseases	18 (19.6%)	6 (11.1%)	12 (31.6%)	0.02^***^
Report the aborted case to a veterinarian	75 (81.5%)	38 (70.9%)	37 (97.4%)	<0.01^***^
Eating cow placental debris (***EAT***)^2)^	10 (10.9%)	2 (3.7%)	8 (21.1%)	0.01^***^
**Average**	39.1%	28.6%	49.5%	0.04^***^
**Practice (P)**				
Self-removal of placental debris by bare hand (Yes) (***HAND***)^2)^	60 (65.2%)	42 (77.8%)	18 (47.4%)	<0.01^***^
Separate diseased animals from others (Yes) (***SEPARATE***)^2)^	46 (51.1%)	23 (42.6%)	23 (60.5%)	0.09^**^
Separate animals that have aborted from others (Yes)	39 (42.4%)	19 (35.2%)	20 (52.6%)	0.10^*^
Sell milk from cows that have aborted (Yes)	22 (23.9%)	0 (0.0%)	22 (57.9%)	<0.01^***^
Consume milk from cows that have aborted (Yes)	17 (19.4%)	0 (0.0%)	16 (42.1%)	<0.01^***^
** ** **Average**	41.6%	31.1%	52.1%	0.25

1) Following questions estimate the knowledge of Brucellosis if farmers answer “Known” or “Have heard” about Brucellosis. In parentheses “True” is the correct answer and “False” is the incorrect answer.

2) Bold text in parentheses represents the variables used in the Probit model (Table 3).

***, **, and * Indicate significance at the 1%, 5%, and 10% levels, respectively.

**Table 3:** Social and farm factors affecting *Brucella* seropositivity at farm level

*Brucella* seropositivity (Probit)	Coefficient	Robust std. error.	P>z
Farmer age (*AGE*)	0.019	0.030	0.523
Education (*EDU*)	0.285	0.636	0.654
Experience (*EXP*)	−1.027	0.852	0.228
Cattle herd size (*CATTLE*)	0.075	0.030	0.011^**^
Raising system (*RAISE*)	30.899	1.729	<0.01^***^
Common Grazing (*GRAZING*)	31.359	1.550	<0.01^***^
Training (*VET TRAIN*)	−4.626	0.515	<0.01^***^
Know brucellosis disease (*KNOW*)	−0.786	0.866	0.364
Self-removal of placental debris by bare hand (*HAND*)	6.406	0.723	<0.01^***^
Separate diseased animals from others (*SEPARATE*)	−8.482	1.358	<0.01^***^
Eating cow placental debris (*EAT*)	4.035	0.687	<0.01^***^
Abortion case (*ABORTION*)	−0.115	0.377	0.761
_cons	−13.087	2.003	0.000

***, **, and * Indicate significance at the 1%, 5%, and 10% levels, respectively.
